# In-Depth Study of Laser Diode Ablation of Kapton Polyimide for Flexible Conductive Substrates

**DOI:** 10.3390/nano8070517

**Published:** 2018-07-11

**Authors:** Francisco J. Romero, Alfonso Salinas-Castillo, Almudena Rivadeneyra, Andreas Albrecht, Andres Godoy, Diego P. Morales, Noel Rodriguez

**Affiliations:** 1 Pervasive Electronics Advanced Research Laboratory (PEARL), Department of Electronics and Computer Technology, University of Granada, 18071 Granada, Spain; agodoy@ugr.es (A.G.); diegopm@ugr.es (D.P.M.); noel@ugr.es (N.R.); 2 Department of Analytical Chemistry, University of Granada, 18071 Granada, Spain; alfonsos@ugr.es; 3 Institute for Nanoelectronics, Technical University of Munich, 80333 Munich, Germany; almudena.rivadeneyra@tum.de (A.R.); andreas.albrecht@tum.de (A.A.)

**Keywords:** laser-induced graphene, polyimide, flexible electronics, laser-scribing, sheet resistance, contact resistance

## Abstract

This work presents a detailed study of the photothermal ablation of Kapton^®^ polyimide by a laser diode targeting its electrical conductivity enhancement. Laser-treated samples were structurally characterized using Scanning Electron Microscopy (SEM), Raman spectroscopy, X-ray Photoelectron Spectroscopy (XPS), as well as Diffuse Reflectance Infrared Fourier Transform (DRIFT) spectroscopy. The results show that the laser-assisted ablation constitutes a simple one-step and environmental friendly method to induce graphene-derived structures on the surface of polyimide films. The laser-modified surface was also electrically characterized through the Transmission Line Method (TLM) aiming at the improvement of the conductivity of the samples by tuning the laser power and the extraction of the contact resistance of the electrodes. Once the laser-ablation process is optimized, the samples increase their conductivity up to six orders of magnitude, being comparable to that of graphene obtained by chemical vapor deposition or by the reduction of graphene-oxide. Additionally, we show that the contact resistance can be decreased down to promising values of ∼2 Ω when using silver-based electrodes.

## 1. Introduction

Carbon nanostructures and graphene-derived sheets are auspicious materials for different areas in science and technology, such as healthcare, flexible electronics or energy storage [1,2]. Nevertheless, reproducible cost-effective methods for the mass production of samples, which would allow for introducing them into the market, are still being sought. The difficulties associated with the current production methods have raised researchers’ interest in a wide spectrum of techniques to synthesize graphene-based materials. Some of those techniques, such as the epitaxial growth on Silicon Carbide or the Chemical Vapor Deposition (CVD), follow a bottom-up approach pursuing the synthesis of high-quality graphene from a physico-chemical assembly of elemental carbon sources [3,4,5]. However, up to date, they suffer from relatively low yield and high production costs as compared with certain top-down approaches [6,7]. In this work, we revisited one of those top-down production approaches by proposing the carbon-rich aromatic polyimides, such as Kapton^®^ films, as raw-material to obtain inexpensive graphene-derived sheets which, regardless of their lower crystallographic quality, are capturing growing niches of interest [8].

Since its commercialization, Kapton^®^ films have been used in an impressive wide range of applications, highlighting their use as substrate for flexible electronics [9,10,11]. Previous works have reported that the carbon bonds which constitute Kapton^®^’s structure can be partially isolated using charged-particle excitation [12], ultraviolet (UV) laser radiation [13], pyrolysis processes [14] or heat-treatments [15]. However, although all these techniques report a substantial increase of the electrical conductivity, they do not fully satisfy the requirements for a cost-effective production of samples due to their poor spatial resolution or the need for using masks to pattern the polyimide surface [16,17].

The laser assisted production of graphene-derived sheets has been previously applied to materials such as graphene oxide (GO) [18,19], but also to Kapton^®^ polyimide [20,21,22]. This approach offers the advantage of defining high-precision laser-induced graphene (LIG) patterns on the polyimide surface without affecting the unexposed areas, and, therefore, without the need of lithographic masks. This technique is also compatible with roll-to-roll methods, enabling an inexpensive mass-production of samples. In the present work, we further explore the possibilities of this procedure conducting an exhaustive study covering both the structural and electrical characterization. In contrast to previous works, we used a low-power continuous laser diode driven by a Computer Numerical Control (CNC) unit, rather than CO${}_{2}$ lasers, for the photothermal ablation of Kapton^®^ polyimide. The feasibility of this system to induce porous graphene from the bare polyimide substrate has been demonstrated by Scanning Electron Microscopy (SEM), as well as Raman and X-ray Photoelectron Spectroscopy (XPS). In addition, Diffuse Reflectance Infrared Fourier Transform (DRIFT) results confirm that the carbon atoms that compose the 3D structure of the LIG layer result mainly from the removal of carbonyl, phenyl ether and imide bonds from the carbon aromatic rings of the pre-ablated chemical structure of the Kapton^®^ polyimide. The changes in the structure have also been studied as a function of the level of ablation (laser power), seeking the enhancement of the electrical conductivity of the samples. Finally, the contact resistance for different electrode materials has been studied as a key parameter for electronics applications.

## 2. Materials and Methods

### 2.1. Materials

Kapton^®^ HN polyimide films with a thickness of 125 μm from DuPont^TM^ were used as raw material in these experiments. This polyimide, which is produced from the condensation of pyromellitic dianhydride with 4,4-Oxydianiline as cross-linking agent, exhibits an excellent balance of physical, chemical and electrical properties over a wide temperature [23]. Ag-loaded conductive paint (from RS, Corby, UK), Carbon-based resistive paste (from Henkel, Dusseldorf, Germany), screen printable AgCl paste (from Henkel) and Graphene Oxide, GO, (from Graphenea, Gipuzkoa, Spain) were also employed as printable contacting electrodes for the electrical characterization of the treated surface.

### 2.2. Exposure Source

**Figure 1:**
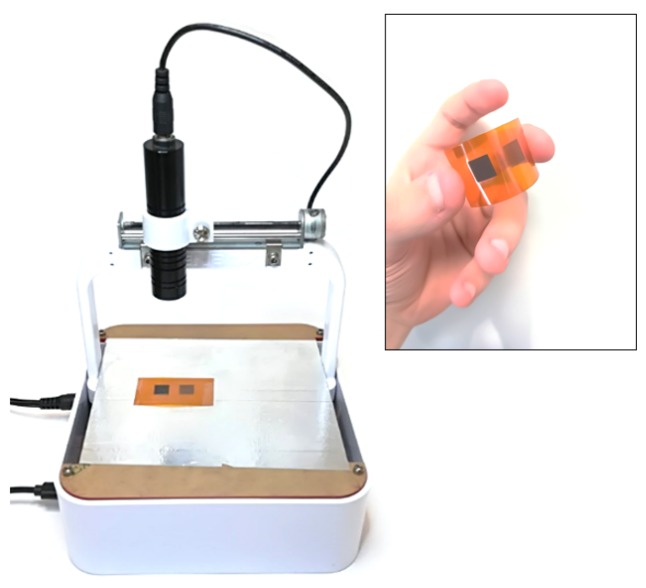
Experimental setup for the laser irradiation of the polyimide surface. The inset displays a sample with two patterned areas of 1 cm${}^{2}$ on the surface scribed at two different values of the laser power: 50 mW (lighter one) and 100 mW (darker one).

The laser-ablation experiments were performed using an in-house developed CNC-driven continuous laser diode (from Q-BAIHE^TM^, model 405ML-300-2290, Shenzhen, China). This system, shown in Figure 1, allows for patterning the polyimide surface, located in an horizontal holder at a distance of 6 cm from the laser head, with a spatial resolution of about 20 μm (given by the mechanical setup) at a fixed wavelength of 405 nm. Furthermore, the laser power can be modulated from 15 mW up to 300 mW.

### 2.3. Structural Characterization

The laser-ablated surfaces were structurally characterized by Scanning Electron Microscopy (SEM), Micro-Raman spectroscopy, X-ray Photoelectron Spectroscopy (XPS) and Infrared (IR) spectroscopy.

SEM-images were recorded with a field-emission scanning electron microscope (NVision40 from Carl Zeiss, Oberkochen, Germany) at an extraction and acceleration voltage of 5 kV. A dispersive micro-Raman spectrometer (JASCO NRS-5100, Easton, PA, USA) with a green diode (Elforlight G4-30; Nd:YAG, λ = 532 nm) as excitation source was used for the Raman spectra acquisition. The XPS experiments were carried out on a Kratos Axis Ultra-DLD (Manchester, UK), using an X-ray (Al Kα, hv =1486.6 eV) power of 450W in a vacuum chamber where the pressure was kept below $10^{-10}\;Torr$. On the other hand, the standard IR transmission spectroscopy is not sensitive enough for our purpose due to the thickness of the Kapton^®^ samples, as reported by previous works [24]. For this reason, we selected the DRIFT spectroscopy, which is well-known for its high sensitivity [25]. Thus, the ablated Kapton^®^ foil was scratched using a scalpel, obtaining a powder (∼2 mg) that was mixed with ∼200 mg of anhydrous KBr powder and pressed into 7 mm diameter discs for its analysis on a Bruker Tensor 27 spectrometer (Billerica, MA, USA).

### 2.4. Electrical Characterization

**Figure 2:**
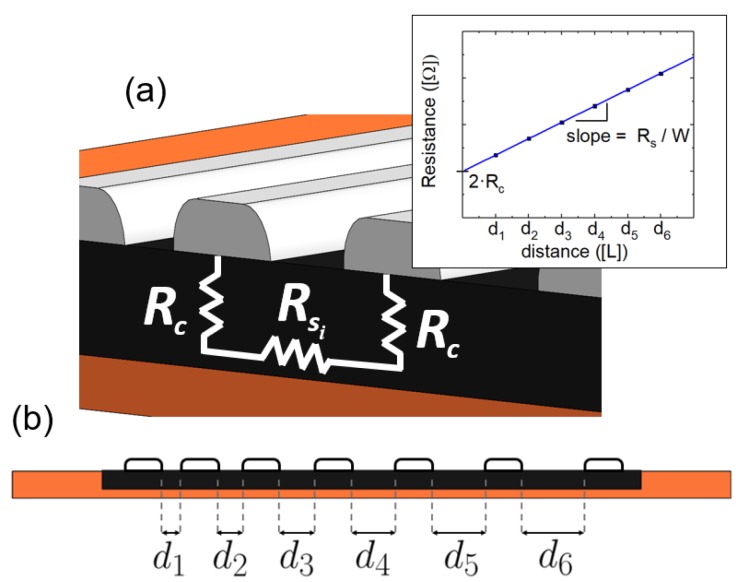
(**a**) equivalent Transmission Line Method (TLM) electrical circuit under two consecutive printed electrodes ($R_{c}$: contact resistance, $R_{si}$: total resistance between contacts *i* and i+1). The inset shows a model of the total resistance ($R_{T}$) as a function of the distance between consecutive lines and its relation with the ablated Kapton^®^ sheet resistance ($R_{S}$) and contact resistance ($R_{c}$); (**b**) printed contacts are positioned over the modified Kapton^®^ surface at a distance $d_{i}$ from each other, where $d_{i}$ ranges from $d_{1}$ = 1 mm to $d_{6}$ = 3.5 mm in 0.5 mm steps.

The second objective of this work is the study of the modification of the electrical conductivity of the laser-ablated area. One of the fastest and technologically simplest ways to obtain the electrical characterization of the modified Kapton^®^ films is the Transmission Line Method (TLM) [26]. Therefore, once the Kapton^®^ surface was laser ablated, electrical contacts were printed on the polyimide surface at a distance $d_{i}$ from each other, as shown in Figure 2a. The total resistance, $R_{T}$, between two consecutive pads is defined by the following expression [27]:

(1) $$R_{T}=\frac{R_{s}\cdot d_{i}}{W}+2R_{c},$$

$R_{s}$ being the sheet resistance of the sample and *W* the contact width. The relationship of Equation (1) for a generic sample is plotted in Figure 2a (inset), illustrating that the sheet resistance, $R_{s}$, can be obtained from the slope of the linear fit of the total resistance between contacts (measured in our experiments with a Keysight 34461A Digital Multimeter, Santa Rosa, CA, USA) as a function of their interspacing. The contact resistance, $R_{c}$, is evaluated from the residual resistance (d=0, interception of the plot with the total resistance axis), being independent of the contacts’ separation [28,29].

The sheet resistance was evaluated as a function of the laser-ablation level on the Kapton^®^ surface. Accordingly, the samples were prepared following the setup illustrated in Figure 2b, where $d_{i}$ ranges from $d_{1}$ = 1 mm to $d_{6}$ = 3.5 mm in 0.5 mm steps. The laser power was increased from 65 mW to 100 mW (λ = 405 nm, continuous wave) at a fixed excursion rate of 3 min/cm${}^{2}$.

## 3. Results and Discussion

### 3.1. Structural Characterization of Ablated Kapton

**Figure 3:**
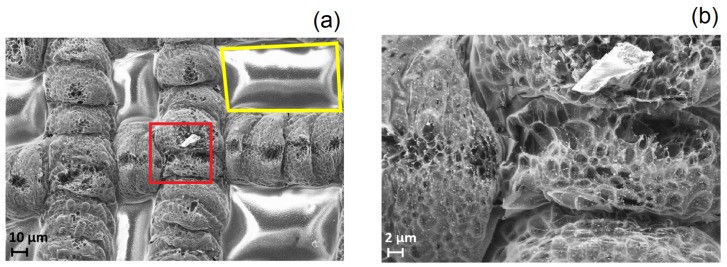
(**a**) SEM image of Kapton^®^ polyimide (scale: 10 μm, extraction and acceleration voltage: 5 kV, working distance: 6.0 mm) ablated using a laser power of 100 mW. The bright areas (such as the one framed in yellow) correspond to the non-irradiated surfaces; (**b**) SEM image (scale: 2 μm, extraction and acceleration voltage: 5 kV, working distance: 6.0 mm) of the red framed area in (**a**) where the porous structure, resulting from the laser ablation process, can be appreciated.

Two SEM micrographs of the irradiated Kapton^®^ surface using a laser power of 100 mW are shown in Figure 3. Irradiated and unirradiated areas can be clearly distinguished: the bright ones correspond to the non-ablated Kapton^®^(yellow box in Figure 3a), while the dark ones are areas resulting of the laser ablation (red box in Figure 3a and corresponding magnification in Figure 3b). The reticulated pattern of the scribing process shown in Figure 3a is a direct consequence of the mechanical step of our CNC unit, which is larger than the laser spot focused on the surface. The sample is laser-scribed in two passes (one for *x* and one for *y*-axis). This fact limits the effective LIG surface of the ablated area, and therefore the minimum value of sheet resistance that can be achieved. The alteration of the color of the Kapton^®^ is visible at a glance: the initial orange tone becomes darker when the laser power is increased, as shown in Figure 1. Futhermore, as seen in Figure 3b, the ablated region exhibits a homogeneously porous foam-like appearance, which indicates a drastic increase in the atomic percentage of carbon [30]. This 3D foam-like morphology is also present in other graphene-based materials with high electrical conductivity such as CVD-grown graphene foams (GFs) presented by Chen et al. [31] or the pure-rGO foams obtained from GO-coated-PU-foams proposed by Samad et al. [32]. This kind of structure makes the ablated layer easily peelable; however, this could be solved by using a thin lacquer-like layer of polymer to cover it, as it is done for Printed Circuit Boards (PCBs).

**Figure 4:**
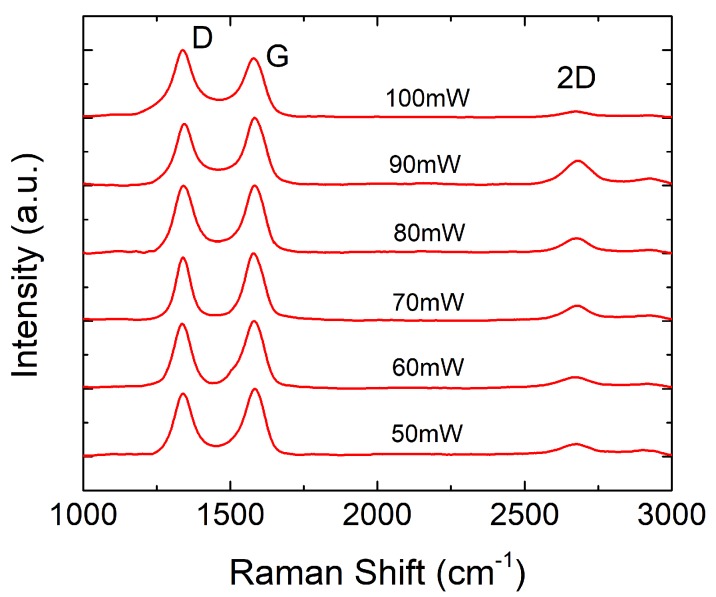
Raman spectra acquired from the laser-treated Kapton^®^ for different laser powers (wavelength: 532 nm, data interval: 1 cm${}^{-1}$, exposure time: 15 s, accumulations: 5, center number: 1469.99 cm${}^{-1}$).

**Figure 5:**
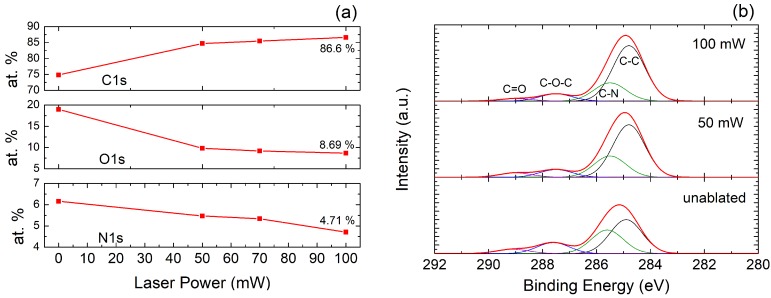
(**a**) atomic percentage of carbon, oxygen and nitrogen in the laser-ablated area as a function of the laser power; (**b**) comparison of the C1s peaks (black: C−C, green: C−N, blue: C−O−C, purple: C = O) from the XPS spectrum of the laser-ablated Kapton^®^ surface using different laser powers (scanned area: 300 × 700 μm${}^{2}$, pass energy: 40 eV, sampling depth: 10 nm, step: 1 eV).

Raman spectroscopy is a very helpful technique for the characterization of these kind of samples in a non-invasive way. The Raman spectrum of the treated surface provides a large amount of information about its structure, as well as a quantification of the disorders and defects introduced by the ablation process [33]. Figure 4 presents the Raman spectra of the laser-ablated surface using different laser photothermal powers in the range from 50 mW to 100 mW. From our experiments, 50 mW was the minimum power to ensure surpassing the ablation threshold for the selected excursion rate (3 min/cm${}^{2}$) [34]. The laser incidence on the Kapton^®^ surface creates two main peaks in the Raman spectra, the so-called D and G peaks, located at ∼1350 cm${}^{-1}$ and ∼1580 cm${}^{-1},$ respectively. These peaks are present in disordered graphitic materials. In particular, the D peak reveals the presence of defects in $sp^{2}$-hybridized carbonous systems [35], while the G peak indicates a graphite-derived structure. The 2D peak (∼2700 cm${}^{-1}$) is due to a second-order resonance, and gives information about the number of layers of the graphite structure [36]. As observed in Figure 4, the ratio $I_{D}/I_{G}≃1$ for a laser power below 100 mW confirms the crystalline nature of the ablated surface [37], whereas the increase of the $I_{2D}/I_{G}$ ratio indicates a reduction of the number of graphene layers [38]. The resulting Raman spectra of the LIG for the higher laser powers (70,80,90) mW are similar to the one in nanographene [33]. However, once the laser power exceeds 90 mW, the surface begins to deteriorate and then both $I_{2D}/I_{G}$ and $I_{G}/I_{D}$ ratios decay. At this point, the surface is mainly composed of $sp^{2}$ amorphous carbon [39]. The laser-treated films were further characterized by XPS analysis. The XPS data demostrate that the ablated surface is mainly composed of carbon, oxygen and nitrogen, whose atomic contents as a function of the laser power are illustrated in Figure 5a. The results obtained show that the laser is able to break the C−N, C−O−C and C=O bonds of the untreated Kapton^®^ films, entailing a drastic increase in the atomic content of carbon, while both oxygen and nitrogen are released as gases as a consequence of the laser irradiation [40]. The lower decay of nitrogen ratio suggests the formation of intermediate nitrile groups (−C≡N) [25]. As a result of the rapid removal of some $sp^{2}$ carbon atoms from the plane, as well as a great part of the bonds of carbon with both nitrogen and oxygen, the ablated surface adopts the porous morphology that can be appreciated in Figure 3, which is a characteristic of the LIG.

Figure 5b shows the high resolution XPS C1s spectra for different laser powers using a Gaussian decomposition resolved with the CasaXPS software (version 2.3.15, Casa Software Ltd, Teignmouth, UK). All of the spectra are calibrated with respect to the carbon peak (284.6 eV). The spectrum of the Kapton^®^ film presents mainly four functional groups: carbon in the aromatic rings (C−C), C−O−C bonds, carbon bonded to nitrogen (C−N) and carbonyls (C=O) [41,42,43]. As expected, the photothermal process implies significant modifications in the C1s peak intensity and its shape. These results confirm that, as the laser power increases, the number of C=O, C−O−C and C−N bonds decreases, while the C−C peak increases, which is in agreement with the atomic percentages obtained.

**Figure 6:**
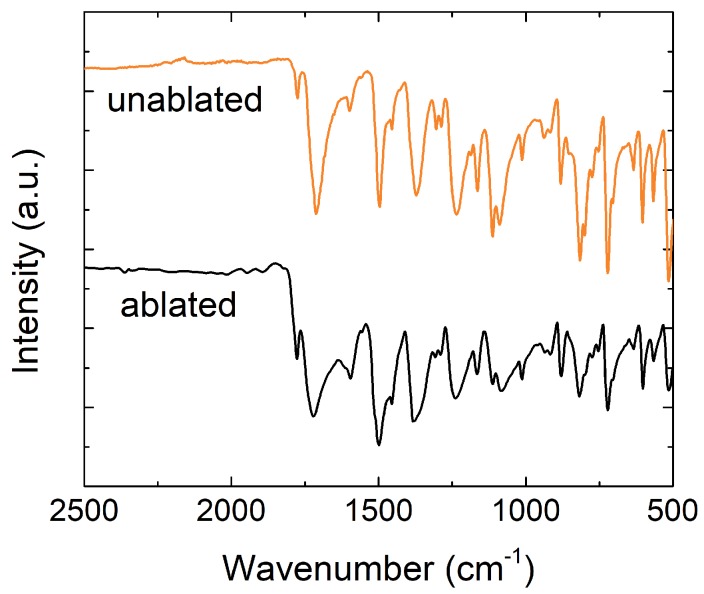
Diffuse Reflectance Infrared Fourier Transform (DRIFT) spectra of Kapton polyimide before and after the laser irradiation (number of scans: 1024, resolution: 1 cm${}^{-1}$).

DRIFT spectroscopy also indicates the removal of these bonds. Figure 6 shows the spectra of the unablated and ablated areas of the Kapton^®^ polyimide with its characteristic absorption bands, whose assignment is given in Table 1. To establish an appropriate comparison, both curves are baseline calibrated with respect to the intensity of the spectrum acquired in the range (1800–2500) cm${}^{-1}$, which is the least influenced by the laser diode ablation [24]. As can be seen, the spectrum of the ablated area presents a significant reduction of the aromatic C−H stretching modes, the imide C−N peaks as well as the phenyl ether linkage (C−O−C), whereas the intensities of the 1500 cm${}^{-1}$ and 1600 cm${}^{-1}$ peaks reveal an increase in the aromaticity of the ablated surface.

### 3.2. Electrical Characterization of Ablated Kapton

**Figure 7:**
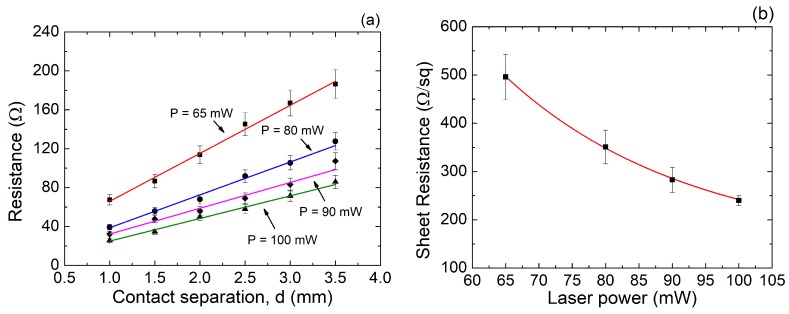
(**a**) total resistance extracted from the Transmission Line Method (TLM) using Ag electrodes; (**b**) sheet resistance as a function of the laser power at an excursion rate of 3 min/cm${}^{2}$.

The experimental values of the total resistance between contacts using Ag electrodes, and extracted from a TLM structure for different values of the laser ablation power, are presented in Figure 7a. As expected, the total resistance exhibits a linear dependence with respect to the electrodes’ separation [45]. Additionally, all curves converge to the same point (±14Ω) in the ordinate axis, indicating that the contact resistance is almost independent of the laser power. The sheet resistance of the LIG, extracted from the slope of the linear fit, is plotted in Figure 7b as a function of the laser power. The boost of the conductivity is correlated with the ablation level of the surface, and therefore with the aromaticity of the samples. In the same way that the atomic percentages of carbon change, the sheet resistance presents a nonlinear behavior with respect to the laser power. As observed, the decrease of the sheet resistance tends to saturate at $∼250\;\Omega \;sq^{-1}$; a value that could be lowered by uniformly patterning the ablated area (instead of using a reticulated ablation mesh as shown in Figure 3) [20]. Even so, as detailed in Table 2, this value is very competitive with respect to the obtained from other graphene-based sheets whose synthesis process is technologically more complex, such as occurs in chemical methods.

In addition to the sheet resistance, the quality of the electrical contact between the ablated surface and the access electrodes is a crucial aspect for the development of high-performance devices [46]. The contact resistance was measured by the TLM method for different electrode materials (Ag, AgCl, rGO and a carbon-based paste). The results obtained are presented in Table 3. Silver and carbon-based contacts were printed on the ablated surface with the pattern presented in Figure 2b, while the rGO contacts were fabricated from the laser-assisted reduction of a GO electrode over the Kapton^®^ ablated surface [47]. We report promissing contact-resistance values, $R_{c}$, as low as 2 Ω for the Ag and AgCl electrodes, while the reduced-GO and the carbon resistive paste ones yield a contact resistance of ∼75Ω and ∼125 Ω, respectively. These contact resistance values are much lower than the obtained for others’ graphene-derived sheets [48]; this fact might be correlated with the great porosity of the laser-ablated Kapton^®^, which enhances the specific contact area of the printed electrodes.

The results obtained from both sheet resistance and contact resistance reflect the great potential of the laser diode ablation of Kapton^®^ polyimide films, since it constitutes an inexpensive, simple and environmentally friendly method to generate conductive graphene-derived patterns on flexible substrates, anticipating applications in the field of biosensors [52,53] and energy storage [19].

## 4. Conclusions

The laser diode ablation of Kapton^®^ polyimide has been demonstrated as a high-precision method to modify its surface-structure and electrical properties. The laser-treated surface has been structurally characterized by SEM, Raman, XPS and DRIFT spectroscopy. The results obtained validate the capability of the laser diode photothermal process to induce porous graphene on the polyimide surface. Furthermore, the electrical conductivity has been studied as a function of the laser power aiming at the optimization of the resistivity of the samples. The experiments have shown promising values of electrical conductivity ($R_{s}∼250\;\Omega \;sq^{-1}$), once the laser-assisted photothermal process has been tuned. The results have also shown independence of the contact resistance with the laser power, presenting values as low as 2 Ω for Ag-based electrodes. These outcomes pave the way for the application of this eco-friendly and low-power manufacturing technology in the flexible electronics field.

## Figures and Tables

**Table 1:** Infrared absorption bands’ identification [44].

Bond	Wavenumber (cm${}^{-1}$)
C=O (Carbonyl)	1775, 1712, 1165
C−C (Aromatic)	1600, 1500
C−N (Imide)	1371, 1305, 1285, 1112, 1088
C−O−C (Aromatic)	1235
C−H (Aromatic)	1012, 937, 880, 815
C−H or C−N	721

**Table 2:** Comparison of the sheet resistance, $R_{s}$, extracted from close related graphene-derived samples.

	Kim et al. [49]	Li et al. [50]	Zhao et al. [51]	Romero et al. [47]	Lin et al. [20]	This Work
Sample	Graphene/PET	Graphene/SiO${}_{2}$	rGO/PET	rGO/PET	LIG/Kapton	LIG/Kapton
$R_{s}$ (Ω/sq.)	280	350	840	226	<35	[242, 295]
Method	CVD	CVD	chemical reduction	laser diode	CO${}_{2}$ laser	laser diode

**Table 3:** Values of the contact resistance, $R_{c}$, extracted from different material-based electrodes.

Electrode Material	Contact Resistance (Ω)
Ag, AgCl	2 (±6.5%)
Laser-rGO	75 (±30%)
Carbon-based paste	125 (±20%)
